# What’s the Matter? Alcohol Use Risk Among Relatives of People with Mental Illness

**DOI:** 10.3390/ijerph21121637

**Published:** 2024-12-09

**Authors:** Suzanne A. McKeag, Gordon L. Flett, Joel O. Goldberg

**Affiliations:** Department of Psychology, York University, Toronto, ON M3J 1P3, Canada; gflett@yorku.ca (G.L.F.); jgoldber@yorku.ca (J.O.G.)

**Keywords:** family burden, caregiving, alcohol, social support, hopelessness, mattering, well-being

## Abstract

Family members who live with relatives with serious mental illness face unique mental health risks, which become worse with alcohol use and without social support. Research has highlighted the damaging effects of harmful substance use among people who feel like they do not matter to others, but few studies have assessed links between mattering and alcohol use within marginalized populations. In the present study, a sample of family members who reside with a relative with mental illness completed an online survey. Using the AUDIT alcohol screening measure, participants were classified into a No–Low Risk Alcohol Use (*n* = 52) or a Hazardous Drinking (*n* = 28) group. Hazardous alcohol use was alarmingly high, reaching triple the rate of the general population and categorized at the most severe level of harm. Those who drank hazardously felt like they mattered less to others (*p* < 0.001), felt like they mattered less to their relative with mental illness (*p* = 0.035), had greater anti-mattering (e.g., they felt invisible and unheard) (*p* = 0.008), experienced more hopelessness (*p* < 0.001), felt less supported by significant others (*p* = 0.003), endorsed having more problems with mental health services (*p* = 0.017), had higher stigma (*p* < 0.001), and had lower psychological well-being (*p* < 0.001). Findings highlight under-recognized public health risks, implications for public health initiatives, and the need for tailored interventions that boost mattering and reduce harmful alcohol use in this vulnerable family member population.

## 1. Introduction

The global burden of mental health disorders has had profoundly negative impacts on not only those who experience the conditions themselves [1] but also upon their families [2,3,4,5]. These difficulties are commonly referred to as family burden, and they include experiences that are both objective, such as caregiving demands, and subjective, such as psychological distress [6]. Family members typically experience role changes and disruption in routines and home life, and there can be a serious emotional toll on their sense of well-being [7]. Some research has found greater burden among those who live in the same home as a relative with mental illness [8,9], including worse experiences in providing caregiving [10] and less social support, heightening these family members’ feelings of isolation and marginalization [11]. A study by Van der Sanden et al. [6] examined the impacts of strategies used to cope with family burden and found that maladaptive approaches, including substance use, increased psychological distress among relatives of people with mental illness. However, there is a dearth of research on substance use in this population [2], despite its known public health implications [12], and remarkably few studies that have examined how alcohol overuse might be associated with important risk and resilience factors.

Amidst the strain of family burden, seeking social support to cope has been found to benefit individuals by fostering more effective caregiving [5,13], promoting more positive coping, and enhancing their well-being [14]. However, many family members instead withdraw from their social connections and become isolated [2,15], including limiting their contact with friends and romantic partners [16]. Within this socially compromised population, those who reside with relatives with serious mental illness have been identified as having even less social support than those who did not live with their ill family members [11]. The lack of interpersonal connection is especially concerning among relatives of people with mental disorders, as they do not receive the important benefits that social relationships provide, and burden tends to mount and fill this void [5,13,14].

Family members have also coped with the burden by seeking education about mental illness [17] and information about mental health care systems [15]. This psychoeducational approach seems effective in relieving burdensome emotional distress, since having more favourable experiences with mental health care systems has been associated with reduced depressed mood in this family member population [10]. However, many individuals continue to remain insufficiently informed about the disorder of their relative, and thus they feel ill-equipped to truly comprehend their family member’s various needs and adequately care for them [14,18,19]. At the same time, others have felt invalidated and powerless when interacting with mental health care professionals [15]. Siblings, in particular, have not only felt uninformed and overlooked by professionals but also neglected by their own parents, who they have perceived to be immersed in family burden distress and, albeit, perhaps understandably, focused narrowly on their ill child [14]. These experiences have left many family members feeling despair and hopelessness [11,20].

When positive strategies for coping with family burden have been perceived as unavailable or ineffective, some have turned to maladaptive methods of coping. These approaches have provided some temporary relief at times, though at the cost of undesirable impacts on their well-being and on their capacity to act effectively in the long term [6,13,21]. For example, among family members who have relatives with mental illness, substance use was found to significantly mediate the relationship between family burden and psychological distress and between family burden and quality of life, such that substance use was associated with worse outcomes in both cases [6]. Despite these negative consequences, relatives of people with mental illness have turned to substances to escape intolerable emotional distress [6,13,22]. Indeed, relief is sought from various kinds of emotional pain experienced by family members, including depression, anxiety [22], stress [2], guilt, shame, worry, resentment [23], grief [10], anger, fear [4], and helplessness [11].

More broadly, attempts to understand addictive behaviours in families have considered the influence of a construct termed ‘mattering’, and the literature in this area offers a compelling theoretical framework upon which substance use among family members of people with mental conditions may be explored [24,25]. Mattering is a person’s perception that they are paid attention to, valued, relied on [26], and appreciated [27], and their belief that they enhance the value of others [28]. Categories of mattering include general mattering, which is a person’s overall sense of mattering [26]; mattering to others, which is one’s sense that they matter to a specific person [29]; and anti-mattering, which is an individual’s sense that they are not seen or heard by others, so they feel invisible and insignificant [30]. In an examination of mattering and substance use in families, Elliott [24] noted that individuals experience excruciating emotional pain in the aftermath of perceiving that they have had no impact on others and proposed that people may subsequently take extreme actions, such as using substances excessively, to alleviate their anguish. This formulation was supported by evidence summarized in a later review and analysis of mattering and substance use literature by Flett et al. [25], who found that lower levels of mattering and, to a greater extent, higher levels of anti-mattering were associated with immense psycho-social pain and elevated risk of harmful substance use and addictive behaviours. On a related note, Elliott [24] proposed that individuals may believe that, since they are not valued, no one would be affected if they harmed themselves, so indulging in excessive substance use would be inconsequential. Building on this idea, Flett et al. [25] noted that it is improbable that those with low levels of mattering seriously contemplate the negative impacts of substance use, since robust links exist between higher levels of mattering, a greater sense of hope [31], a future orientation, and reduced substance use and problems [32,33]. Moreover, a strong sense of mattering has been found to reduce the likelihood that a person will engage in harmful substance use [25] and to be significantly associated with lower levels of depression, greater perceived purpose in life, and increased overall well-being [34].

Experiencing chronic and frequent marginalization, and thus feeling deeply unimportant, has been associated with a sense of hopelessness and increased harmful substance use [25]. As such, in light of the lack of social support [2,11,15,16], devaluation, and impotence that individuals have felt as they have strived to support relatives with mental illness, it is not surprising that they have also felt invisible [14], which is a central feature of anti-mattering [30], as well as hopelessness [11,20] and distress [2,4,10,22,23], which are negatively correlated with mattering [25]. However, only one study to date has directly examined mattering in this family member population, finding that those who live with a relative with mental illness, versus those who do not, showed a trend in which they felt that their lives mattered less [11].

The goals of the current study were to examine alcohol use in the marginalized population of family members who live with a relative with mental illness and its associated harmful outcomes, especially hopelessness and reduced well-being, consistent with previous findings of substance use as a way of problematic coping with family burden. However, most importantly, we were interested in exploring the formulation that alcohol overuse might be associated with an increased sense of anti-mattering and a reduced sense of mattering, and how these relate to their caregiving and their social supports in this disadvantaged family member population.

## 2. Materials and Methods

### 2.1. Participants

Participants were recruited, as part of a larger study on stigma and serious mental illness [11], through family support services at the Canadian Mental Health Association, the Institute for Advancements in Mental Health, and Reconnect Community Health Services. The organizations emailed a recruitment advertisement to clients who had accessed their services and consented to receive their communications. Additional participants were recruited through the Schizophrenia Society of York University (SSY), an organization that promotes schizophrenia awareness, which posted the recruitment advertisement on their Instagram account.

A total of 254 people indicated that they had a relative with a mental illness, including disorders such as schizophrenia or bipolar and excluding neurocognitive conditions such as dementia. Invalid profiles (*n* = 8), those with missing data (*n* = 123), and those belonging to people who did not live with a relative with mental illness (*n* = 43) were removed from the dataset, resulting in a final sample of 80 people. With regard to categories of family relationships, participants were mainly parents, siblings, adult children, and spouses, with the exception of one niece and one stepmother.

### 2.2. Study Design and Sample Characteristics

Based on the Alcohol Use Disorders Identification Test (AUDIT, [35]) self-reported drinking behaviour scores, family members were divided into No–Low Risk (*n* = 52) and Hazardous Drinking (*n* = 28) groups, which were compared on the measures described below. Demographics for each group of participants are shown in Table 1. Members of the No–Low Risk group ranged in age from 18 to 80 years old; their mean age was 51.9 years (*SD* = 15.2). Those in the Hazardous Drinking group ranged in age from 21 to 67 years old; their mean age was 29.1 years (*SD* = 8.1). The No–Low Risk group was significantly older than the Hazardous Drinking group on average (*t*(76.98) = 8.70, *p* < 0.001). The age difference appears to be accounted for by the predominance of parents in the No–Low Risk group compared to the Hazardous Drinking group (63%, *n* = 33/52 versus 7%, *n* = 2/28), and to the predominance of adult children and siblings in the Hazardous Drinking group compared to the No–Low Risk group (children: 25%, *n* = 7/28 versus 8%, *n* = 4/52; siblings: 39%, *n* = 11/28 versus 19%, *n* = 10/52). Additionally, compared to the No–Low Risk group, the Hazardous Drinking group had more spouses (29%; *n* = 8/28 versus 6%; *n* = 3/52), fewer women (36%, *n* = 10/28 versus 81%, *n* = 42/52), and fewer White people (57%, *n* = 16/28 versus 71%, *n* = 37/52).

See Table 2 for demographics of participants’ relatives. The mean age of the relatives was not significantly different between the groups (*t*(41.69) = −0.05, *p* = 0.957, *d* = 0.01). Upon inspection, the distributions of gender and race/ethnicity appeared to be similar, but the clinical diagnoses of relatives’ mental illnesses were unevenly distributed between the groups. Most notably, in the Hazardous Drinking group, the most frequently reported condition was Other Psychotic Disorders (82%, *n* = 23/28), followed by Schizophrenia (46%, *n* = 13/28), and no participant identified their relative as having an Other Mental Disorder (0%, *n* = 0/28). In contrast, in the No–Low Risk group, the most frequently reported condition was Schizophrenia (35%, *n* = 18), followed by Other Mental Condition (31%, *n* = 16). Note that percentages of disorders exceed 100% because each participant could report multiple mental health conditions, as concurrent disorders are common, and to align with reporting in previous research on relatives of people with mental illness [6].

### 2.3. Measures

Alcohol use was measured by the 10-item Alcohol Use Disorders Identification Test (AUDIT): Self-Report Version (AUDIT, [35]). Eight AUDIT questions, including “How often do you have a drink containing alcohol?”, were answered by selecting one of five responses ranging from zero to four, with zero representing the lowest and four representing the greatest frequency and volume of alcohol consumption. The other two AUDIT questions asked if and when participants experienced alcohol-related issues, to which individuals responded by selecting either “0 = No”, “3 = Yes, but not in the last year”, or “5 = Yes, during the last year”. AUDIT scores can range from zero to forty; greater scores represent higher-risk drinking. Four levels of alcohol use risk are outlined. Zone I is low risk; scores range from one to seven, and education is recommended to prevent escalated use. Zone II is Hazardous/Harmful Drinking; scores range from eight to fifteen, and advice about reducing alcohol use is recommended. Zone III is the next level of Hazardous/Harmful Drinking; scores range from 16 to 19, and advice about reducing alcohol use, counselling, and monitoring are advised. Finally, Zone IIII is the highest level of Hazardous/Harmful Drinking; scores range from twenty to forty, and referral to a specialist for further assessment and treatment is advised. Following these cut-off guidelines [36], participants who scored eight or higher were assigned to a Hazardous Drinking group and those who scored seven or less were assigned to a No–Low Risk group, which were compared in the present study. Cronbach’s alpha was excellent (α = 0.94).

General mattering was assessed by the 5-item General Mattering Scale (GMS, [37]). Participants responded to GMS questions, such as “How much do other people depend on you?”, by selecting one of four options, from “1 = Not at all” to “4 = A lot”. GMS total scores can range from five to twenty; higher scores represent a greater sense of mattering to others overall. Cronbach’s alpha was uncharacteristically somewhat low (α = 0.57).

Mattering to others was measured by the 11-item Mattering to Others Scale (MTOS, [29]). The original five-point Likert scale was adapted to be on a three-point Likert scale to simplify the response options and make them clearer to participants. As such, participants responded to nine MTOS statements, including “I feel special to my relative with mental illness”, by choosing one of three answers ranging from “1 = not much” to “3 = a lot”. For the additional two MTOS questions, including “If your relative with mental illness made a list of all the things she thinks about where do you think you’d be on the list?”, individuals selected a number from one to five, with “1 = bottom” and “5 = top”; responses of one and two were scored as one point, three and four were scored as two points, and five was scored as three points. MTOS total scores can range from 11 to 33; higher scores indicate a greater sense of mattering to a particular person, who was the participants’ relative with mental illness in the present study. Cronbach’s alpha was good (α = 0.80).

Anti-mattering was measured by the 5-item Anti-Mattering Scale (AMS, [38]). In response to questions, including “To what extent have you been made to feel like you are invisible?”, participants selected one of four ratings, from “1 = Not at all” to “4 = A lot”. AMS total scores can range from five to twenty; higher scores indicate a greater sense of not mattering to others overall. Cronbach’s alpha was acceptable (α = 0.79).

Hopelessness was measured by the 20-item Beck Hopelessness Scale (BHS, [39]). Participants answered “True” or “False” to statements such as “My future seems dark to me”. Responses that endorse hopelessness are scored one, and those that do not are scored zero. Total scores can range from zero to twenty; higher scores represent more hopelessness. Cronbach’s alpha was good (α = 0.85).

Experiences in caregiving were measured by the four subscales of the 19-item Brief Experiences in Caregiving Inventory (BECI, [40]): stigma, positive personal experiences, problems with services, and difficult behaviours. Participants reported how often they thought about aspects of caregiving, such as the behaviour of their relative with mental illness being “unpredictable” or “How to deal with health care professionals”, by selecting one of five responses, from “0 = never” to “4 = always”. BECI subscale scores can range from zero to sixteen for Stigma, from zero to twenty for Problems with Services, and from zero to twenty-four for Difficult Behaviours; higher scores on these scales represent more challenging experiences in caregiving. Positive Personal Experiences scores can range from zero to sixteen; higher scores represent more favourable experiences in caregiving. Cronbach’s alpha was moderate for Stigma (α = 0.63), acceptable for Positive Personal Experiences (α = 0.70), acceptable for Difficult Behaviours (α = 0.78), and good for Problems with Services (α = 0.82).

Psychological well-being was measured by the six subscales of Ryff’s Psychological Well-being Scales 42-Item Version (PWBS, [41]): Autonomy, Environmental Mastery, Personal Growth, Positive Relationships, Purpose in Life, and Self-Acceptance. Participants reported their agreement with statements such as “I have a sense of direction and purpose in life” by selecting a number from “1 = Strongly Disagree” to “6 = Strongly Agree”. PWBS subscales have seven items each, and their scores can range from seven to forty-two; higher scores are associated with better psychological well-being. Cronbach’s alpha was good for Self-Acceptance (*α* = 0.80) and Personal Growth (*α* = 0.81), acceptable for Autonomy (*α* = 0.72), Positive Relationships (*α* = 0.72), and Purpose in Life (α = 0.73), and somewhat low for Environmental Mastery (*α* = 0.58).

Perceived social support was measured by the three subscales of the 12-Item Multidimensional Scale of Perceived Social Support (MSPSS, [42]): Significant Others, Friends, and Family. Participants selected one of seven options, from “1 = very strongly disagree” to “7 = very strongly agree”, in response to statements such as “There is a special person who is around when I am in need”. The range of possible scores on each MSPSS subscale is one to seven; higher scores represent more social support. Cronbach’s alpha was excellent for Significant Others (*α* = 0.93) and Family (*α* = 0.90) and good for Friends (*α* = 0.89).

### 2.4. Procedure

The study was conducted according to the guidelines of the Declaration of Helsinki and approved by the York University Human Participants Research Committee (REB Certificate #: e2021-316; 23 September 2021). Participants accessed the study survey online at the Qualtrics XM platform via a digital link, where they gave their informed consent, and then they were administered a demographics questionnaire and the 8 questionnaire measures described above. At the same time, participants responded to additional questionnaire measures and short answer questions as part of a larger ongoing programme of research investigating stigma and serious mental illness. Following their participation in the study, individuals received a $5.00 (CND) coffee shop gift card.

### 2.5. Statistical Analyses

Statistical analyses were conducted in R Studio [43]. Descriptive statistics, Shapiro–Wilk, and Levene’s tests were used to assess assumptions. Due to the small sample size and unevenly distributed demographics between comparison groups, the differences in the measures described above between the No–Low Risk group and Hazardous Drinking group were assessed via Welch’s independent *t*-tests. Additionally, effect sizes were computed using Cohen’s d and reliability was assessed via Cronbach’s alpha.

## 3. Results

See Table 3 for the No–Low Risk group and Hazardous Drinking group descriptive statistics, as well as inferential statistics that assessed differences between the groups.

The seriousness of drinking problems in the sample is evident in the Alcohol Use Disorders Identification Test: Self-Report Version (AUDIT, [35]) scores. The Hazardous Drinking group average was 19.9 (*SD* = 5.35), which is at the very top of AUDIT Zone III and on the brink of Zone IV [36]. Alcohol use prevalence was also extremely elevated, with 88.8% of participants (*n* = 71/80) drinking any amount of alcohol in the past year, and of those who drank, 39.4% (*n* = 28/71) meeting criteria for AUDIT Zone II hazardous drinking [36] or higher. Analysis of the difference in AUDIT results between the comparison groups found that the No–Low Risk group scored significantly lower than the Hazardous Drinking group (*t*(30.04) = −17.19, *p* < 0.001, *d* = 5.19).

Hopelessness levels were disconcertingly high among those who drank hazardously. According to the Beck Hopelessness Scale (BHS, [39]) findings, the Hazardous Drinking group’s mean score of 10.46 (*SD* = 1.90) was distinctly above the clinical cut-off guideline score of 9 that has been associated with serious levels of hopelessness and predictive of suicide risk [44]. Compared to the No–Low Risk group’s mean BHS score of 5.29 (*SD* = 4.82), the Hazardous Drinking group’s score was twice as high and significantly elevated (*t*(73.11) = −6.83, *p* < 0.001; *d* = 1.28).

Mattering was extremely compromised among participants who drank hazardously. While total scores on the general mattering scale (GMS, [37]) typically fall between 15 and 16 (30), which was found to be in line with the No–Low Risk group’s mean score of 15.56 (*SD* = 2.54), the Hazardous Drinking group’s mean score of 13.18 (*SD* = 1.89) was significantly lower than that of the No–Low Risk group (*t*(70.07) = 4.75, *p* < 0.001; *d* = 1.02). With respect to anti-mattering, relative to the usually observed scores of 10 to 11 on the Anti-mattering Scale (AMS, [38]), the No–Low Risk group’s mean score was slightly elevated, at 11.46 (*SD* = 4.03), while the Hazardous Drinking group’s mean score of 13.29 (*SD* = 1.98) was significantly greater than the No–Low Risk group’s AMS scores (*t*(77.53) = −2.71, *p* = 0.008; *d* = 0.53). Mattering to others (MTO, [29]) was also significantly lower in the Hazardous Drinking group versus the No–Low Risk group (*t*(77.52) = 2.14, *p* = 0.035; *d* = 0.43).

Examination of the Brief Experiences in Caregiving Inventory (BECI, [40]) found that the Hazardous Drinking group reported experiencing significantly more Problems with Services (*t*(66.62) = −2.46, *p* = 0.017; *d* = 0.54) and experienced more Stigma (*t*(76.02) = −3.32, *p* = 0.001; *d* = 0.68) compared to the No–Low Risk group. Additionally, there was a trend in which the Hazardous Drinking group reported that they had fewer positive experiences caring for their relative (*t*(60.85) = 1.84, *p* = 0.071; *d* = 0.42) and they indicated that their family member engaged in fewer difficult behaviours (*t*(71.61) = 1.79, *p* = 0.077; *d* = 0.38), although these differences were not significant.

Social support results were mixed. Examination of findings for the Multidimensional Scale of Perceived Social Support (MSPSS, [42]) indicated that the Hazardous Drinking group self-reported having significantly less support from Significant Others (*t*(72.63) = 3.08, *p* = 0.003; *d* = 0.65) compared to the No–Low Risk group. However, there were no differences between the groups on their perceived levels of support from Friends (*t*(56.38) = 1.33, *p* = 0.188; *d* = 0.31) or Family (*t*(69.41) = 0.32, *p* = 0.751; *d* = 0.07).

Lower psychological well-being in all domains among family members who drank hazardously versus those who did not was evident in the results of the assessment of the Psychological Well-Being Scales (PWB, [41]). The Hazardous Drinking group self-reported significantly lower well-being than the No–Low Risk group on all subscales, including Autonomy (*t*(76.09) = 5.43, *p* < 0.001; *d* = 1.11), Environmental Mastery (*t*(75.33) = 4.58, *p* < 0.001; *d* = 0.95), Personal Growth (*t*(76.91) = 8.56, *p* < 0.001; *d* = 1.70), Positive Relationships (*t*(76.95) = 5.97, *p* < 0.001; *d* = 1.19), Purpose in Life (*t*(76.99) = 6.79, *p* < 0.001; *d* = 1.36), and Self-Acceptance (*t*(71.52) = 3.92, *p* < 0.001; *d* = 0.74).

## 4. Discussion

The present study is, to the best of our knowledge, the first to examine caregiving, social support, mattering, hopelessness, and well-being among people who live with relatives with serious mental illness in the context of differences on these variables between those who do and those who do not engage in hazardous alcohol use. Notably, our analyses identified a group of family members who had high levels of hazardous alcohol use, which was accompanied by strong feelings of not mattering and significant hopelessness. Family members in the study who drank hazardously were found to have dangerously elevated average alcohol use scores, at the threshold of the highest level of risk according to the Alcohol Use Disorders Identification Test guidelines, at the point where immediate assessment for alcohol dependence syndrome and treatment are advised [36]. In terms of drinking rates, past-year drinking for the family member sample was reported by 88.8% of participants, compared to 76.5% of the general population in Canada [45]. More importantly, of those who drank in the past year, a striking 39.4% of participants drank hazardously, which is triple the number (13.6%) who met this criterion in the general population in Canada [46]. Clearly, the alarmingly high level of alcohol use in this family member sample is concerning, particularly since this specific marginalized group is largely understudied, and as such, the public health concerns remain mainly unrecognized.

Beyond the sheer seriousness of the dangerous levels of alcohol overuse, a further important finding was that participants who engaged in hazardous drinking felt significantly less important and less appreciated by their relative with mental illness and others in general. Furthermore, those who drank hazardously had a greater sense that they did not matter, would not matter, and that others had made them feel outrightly insignificant and invisible. This empirical finding is consistent with broader evidence indicating that those who have felt lower levels of mattering and higher levels of anti-mattering are most vulnerable to engaging in unhealthy substance use [25]. Moreover, as the first study to assess the relationship between alcohol overuse and mattering among relatives of people with serious mental illness, this novel finding contributes further support to the growing body of literature showing the association between mattering and substance misuse in marginalized groups [25].

Those who drank hazardously reported experiencing significantly higher levels of stigma and significantly more problems with mental health services. Perhaps their perceptions of being judged and discriminated against by others, as well as feeling dismissed or frustrated by mental health care professionals, led these individuals to feel cut off from their capacity to have a positive impact on their relatives and in turn to feel immense distress and hopelessness. These negative experiences and distressing emotions may have contributed to family members’ use of alcohol to detach themselves from their despair, which aligns with both the anguish relief formulation proposed by Elliott [24] and the mattering and substance use review conducted by Flett et al. [25]. The issues here are complex; the pattern of alcohol-related disengagement may be self-protective in one sense but may also hinder one’s capacity to provide the kind of care that makes a significant positive difference in the life of a family member and thus represents a lost opportunity to matter to others and to cultivate hope.

As discussed above, our results indicated that the group of family members who drank hazardously had an exceptionally high mean level of hopelessness as assessed by the BHS. Given that hopelessness is regarded as one of the important indicators of suicidality [47], there are reasons to be quite concerned about how these family members are feeling as they evaluate their current lives and their futures. The risk here is considerable when our results are viewed from a person-focused perspective rather than a variable-focused perspective. People with multiple risk factors (i.e., hazardous drinking, feelings of hopelessness, and feelings of not mattering to others) and few assets (i.e., low sense of well-being and social support) require immediate attention and intervention. Parenthetically, it is important to proactively assess family members given this level of risk.

Notably, of family members who engaged in harmful drinking, only a few were parents; the largest number were siblings, followed by spouses, then adult children of persons with mental disorders. Given that past research has shown that experiences of having a family member with mental illness differ depending on the person’s relationship to the individual who is ill (e.g., parents versus siblings) [3,4], these findings suggest that alcohol use is differentially associated with various relationships to persons with mental disorders.

Differing experiences between relatives may offer insight into the finding in the current research that those who engaged in hazardous drinking felt significantly less supported by significant others. Perhaps this is because the majority of those who did not drink hazardously were parents, who may have already had a significant other, quite possibly the other parent of the ill child, prior to the onset of the mental illness. Thus, with a bond established when burden arose, support may have already been present. In contrast, siblings and adult children are less likely to have had a significant other before their relative became ill, and they may have struggled to make romantic connections while wrestling with burden and worry about how potential partners may react to their family life. This is consistent with reports in past research of people experiencing negative impacts on their romantic relationships as a result of having a sibling with a mental disorder [16,48]. With regard to participants who were spouses, who made up a large portion of those who drank hazardously as well, it seems clear that their significant other, being the person who is ill, was not in a position to support them in their struggles with family burden.

Finally, participants who drank hazardously had significantly lower levels of well-being across all domains, which is consistent with research on alcohol use and well-being [49]. However, well-being has been found to be low among family members of people with mental illness in general [50]. Taken together, these findings suggest that those who drink alcohol hazardously and who live with a relative with mental illness are at an even greater risk for substantially low well-being. It is noteworthy that these deficits in well-being seem to signify that those people who drink hazardously not only suffer from an unmet need to matter, but they also have core frustration in terms of needing to feel positively connected to others, as well as autonomous and competent.

The findings of dangerously elevated alcohol use levels among family members pose serious risks [12] not only to the individuals who drink but also potentially to the mental and physical health of their relatives with mental disorders. Just as family members are impacted by their ill relatives, their alcohol overuse has potential consequences for their relative, thus multiplying this public health risk. Early intervention and prevention are strongly advised and effective in reducing alcohol problems [12], and customized approaches that bolster mattering, particularly within one’s community and culture, have been especially successful within marginalized populations [25,51]. With this in mind, findings from the present study suggest that hazardous drinking reduction initiatives should consider reaching out to people who live with relatives with mental illness with approaches that let them know that they truly matter, which may help to reduce their sense of being invisible to others. By addressing ‘what’s the matter’ among these family members, harmful drinking may be reduced, and positive caregiving experiences and well-being may be increased within families in this vulnerable population.

A limitation was that participants were recruited through organizations that support family members who have relatives with mental illness, which may have resulted in a selection bias. Therefore, it is quite possible that others may be more isolated, that the extent of alcohol problems and negative outcomes is underestimated, and that positive outcomes are fewer in the broader population of people who live with relatives with mental illness. Another limitation is that family members reported the mental illness conditions of their relatives, which is a methodological practice that is consistent with research in this area but leaves the diagnoses not independently verified. In addition, the study sample was somewhat small, some demographics were unevenly distributed, and some variables were not normally distributed, though effect sizes were generally quite good.

Finally, the direction of relationships among variables has been inferred based on relevant literature and theory, yet causal directionality could not be specifically assessed due to the design of the current study and is recommended to explore in future research. In addition, while the current study found higher alcohol use rates and risk in men and younger people consistent with drinking behaviour in the general population in Canada [45], other studies using qualitative methods have identified more women and older adults using alcohol to cope [52]. Furthermore, most participants in the present study were White, and there was an underrepresentation of racial/ethnic diversity in research on family members of people with mental illness. It is recommended that future studies be conducted with a broader and more diverse sample, despite the known recruitment challenges in this population, to better consider differences in patterns of experiences, alcohol use rates and risks, and coping by age, gender, and race/ethnicity, to better understand how to tailor and identify appropriate interventions.

## 5. Conclusions

The findings contribute new insights to the scarce literature on alcohol use among family members who live with people with serious mental illness by identifying dangerously high levels of hazardous drinking compared with the general population. Importantly, evidence was obtained of a key risk factor for those who drank hazardously: they felt like they mattered less to others and as if they were invisible. The individuals who drank hazardously felt more hopelessness, felt less support from significant others, experienced more stigma and problems with services, and had much lower well-being; thus, they appeared to be the most marginalized in this already disadvantaged group. The findings provide evidence for a compelling theoretical framework outlining links between unhealthy substance use and compromised mattering in marginalized populations [25].

Our findings have clear implications for the public health of family members who live with relatives with serious mental illness. Public health initiatives that focus on psycho-education and on tailoring prevention and intervention methods to this population should include messaging and highlight content that serve to bolster their sense of mattering and reduce their sense of being insignificant. Further research with a broader sample will allow for greater recognition of the breadth and depth of the needs and vulnerabilities of family members who live with relatives with serious mental illness.

## Figures and Tables

**Table 1:** Participant demographics.

Demographic	No–Low Risk Group	Hazardous Drinking Group
Participants (*N* = 80) *	*n* = 52	*n* = 28
Age in Years	*M* = 51.9, *SD* = 15.2	*M* = 29.1, *SD* = 8.1
Female	*n* = 42 (81%)	*n* = 10 (36%)
Male	*n* = 9 (17%)	*n* = 18 (64%)
Non-Binary	*n* = 1 (2%)	*n* = 0 (0%)
White/European	*n* = 37 (71%)	*n* = 16 (57%)
BIPOC	*n* = 15 (29%)	*n* = 12 (43%)
Parent	*n* = 33 (63%)	*n* = 2 (7%)
Adult Child	*n* = 4 (8%)	*n* = 7 (25%)
Sibling	*n* = 10 (19%)	*n* = 11 (39%)
Spouse	*n* = 3 (6%)	*n* = 8 (29%)
Other	*n* = 2 (4%)	*n* = 0 (0%)

Note: * *n* = 71/80 participants reported at least some alcohol use in the past year. Note: BIPOC: Black, indigenous, and people of colour.

**Table 2:** Demographics of Relatives with Mental Illness.

Demographic	No–Low Risk Group	Hazardous Drinking Group
Participants (*N* = 80)	*n* = 52	*n* = 28
Age in Years	*M* = 35.8, *SD* = 14.5	*M* = 36.0, *SD* = 11.94
Female	*n* = 17 (33%)	*n* = 12 (43%)
Male	*n* = 32 (62%)	*n* = 16 (57%)
Non-binary	*n* = 3 (6%)	*n* = 0 (0%)
White/European	*n* = 38 (73%)	*n* = 17 (61%)
BIPOC	*n* = 14 (27%)	*n* = 11 (39%)
Schizophrenia	*n* = 18 (35%)	*n* = 13 (46%)
Other Psychotic Disorder	*n* = 11 (22%)	*n* = 23 (82%)
Bipolar Disorder	*n* = 14 (27%)	*n* = 6 (21%)
Major Depression	*n* = 12 (23%)	*n* = 4 (14%)
Other Mental Disorder	*n* = 16 (31%)	*n* = 0 (0%)

Note: BIPOC = Black, indigenous, and people of colour. Note: The sum of the number and percentage of mental illnesses noted is greater than the total number of participants (*N* = 80) because participants reported as many conditions as applied.

**Table 3:** Welch’s Independent *t*-tests comparing scores between the No–Low Risk group and the Hazardous Drinking group.

	N-LR (*n* = 28)		HD (*n* = 28)					
	*M*	*SD*	*M*	*SD*	*M* Difference	*t*-Statistics	*d*	95% CIs
AUDIT: Alcohol Use	2.06	1.72	19.93	5.35	17.9	*t*(30.04) = −17.19, *p* < 0.001 ***	5.19	−19.99, −15.75
GMS: General Mattering	15.56	2.54	13.18	1.89	2.4	*t*(70.07) = 4.75, *p* < 0.001 ***	1.02	1.38, 3.38
MTO: Mattering to Others	23.71	5.27	21.71	3.05	2.0	*t*(77.52) = 2.14, *p* = 0.035 *	0.43	0.14, 3.85
AMS: Anti-mattering	11.46	4.03	13.29	1.98	1.8	*t*(77.53) = −2.71 *p* = 0.008 **	0.53	−3.16, −0.48
Beck Hopelessness Scale	5.29	4.82	10.46	1.90	5.2	*t*(73.11) = −6.83, *p* < 0.001 ***	1.28	−6.69, −3.66
BECI: Stigma	6.62	3.58	8.79	2.25	2.2	*t*(76.02) = −3.32, *p* = 0.001 **	0.68	−3.47, −0.87
BECI: Positive Experiences	10.00	3.11	8.75	2.78	1.3	*t*(60.85) = 1.84, *p* = 0.071	0.42	−0.11, 2.61
BECI: Problems with Services	8.13	4.62	10.46	3.70	2.3	*t*(66.62) = −2.46, *p* = 0.017 *	0.54	−4.22, −0.44
BECI: Difficult Behaviours	13.46	4.90	11.76	3.52	1.7	*t*(71.61) = 1.79, *p* = 0.077	0.38	−0.19, 3.60
PWB: Autonomy	31.06	5.83	25.32	3.55	5.7	*t*(76.09) = 5.43, *p* < 0.001 ***	1.11	3.63, 7.84
PWB: Environmental Mastery	28.12	5.52	23.46	3.49	4.7	*t*(75.33) = 4.58, *p* < 0.001 ***	0.95	2.63, 6.68
PWB: Personal Growth	33.49	6.15	24.46	3.23	9.0	*t*(76.91) = 8.56, *p* < 0.001 ***	1.70	6.93, 11.13
PWB: Positive Relationships	31.31	6.25	24.89	3.31	6.4	*t*(76.95) = 5.97, *p* < 0.001 ***	1.19	4.28, 8.56
PWB: Purpose in Life	31.02	6.34	23.57	3.40	7.5	*t*(76.99) = 6.79, *p* < 0.001 ***	1.36	5.26, 9.63
PWB: Self-Acceptance	28.92	7.64	24.18	2.99	4.7	*t*(71.52) = 3.92, *p* < 0.001 ***	0.74	2.33, 7.16
MSMSS: Significant Other	5.15	1.38	4.34	0.96	0.8	*t*(72.63) = 3.08, *p* = 0.003 **	0.65	0.29, 1.34
MSPSS: Family	4.23	1.58	4.12	1.19	0.1	*t*(69.41) = 0.32, *p* = 0.751	0.07	−0.53, 0.73
MSPSS: Friends	4.71	1.37	4.29	1.35	0.4	*t*(56.38) = 1.33, *p* = 0.188	0.31	−0.21, 1.06

* *p* < 0.05; ** *p* < 0.01; *** *p* < 0.001; Note: No–Low Risk (N-LR); Hazardous Drinking (HD). Note: Alcohol Use Disorders Identification Test (AUDIT); General Mattering Scale (GMS); Anti-mattering Scale (AMS); Brief Experiences in Caregiving Inventory (BECI); Psychological Well-being (PWB); Multidimensional Scale of Perceived Social Support (MSPSS). Note: All significant differences remained following *p*-value corrections conducted via the Benjamini–Hochberg false discovery rate procedure for controlling Type I errors.

## Data Availability

The datasets presented in this article are not readily available because the requesting source must be affiliated with an academic institution. Requests to access the datasets should be directed to the corresponding co-author: jgoldber@yorku.ca.
